# Supplementary material for: A Novel Quantitative Computer-Assisted Score Can Improve Repeatability in the Estimate of Vascular Calcifications at the Abdominal Aorta
Source: Nutrients. 2022 Oct 13;14(20):4276. doi: 10.3390/nu14204276 (PMC9607651; doi:10.3390/nu14204276)

Figure S1. Bland-Altman plots of the intra-operator differences in the estimate of calcification scores between repetitions R1 and R2 (pooled data are presented in Figure 2 in the main text)

Semiquantitative Visual (SV) score

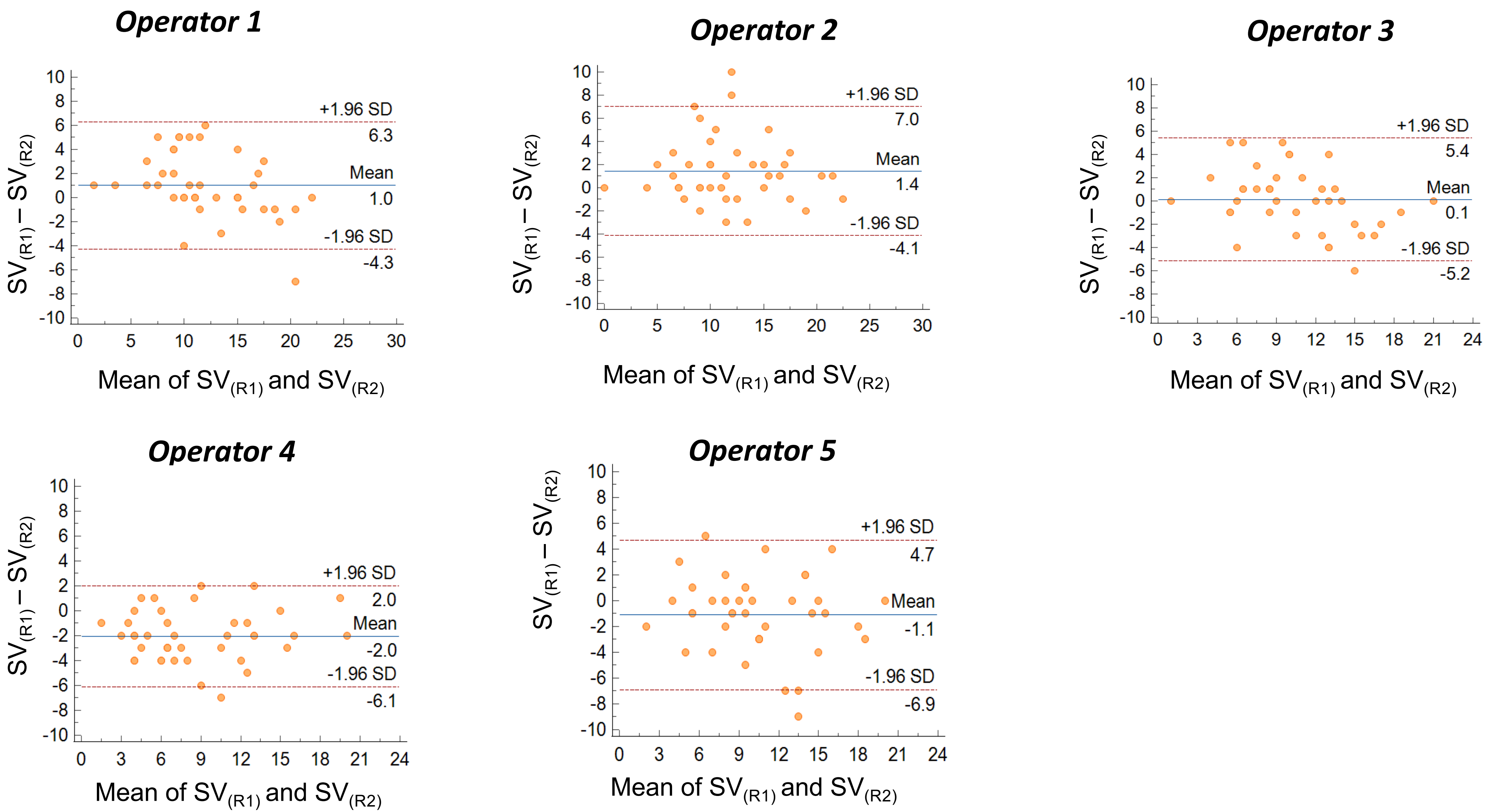

Figure S2. Bland-Altman plots of the intra-operator differences in the estimate of calcification scores between repetitions R1 and R2 (pooled data are presented in Figure 2 in the main text)

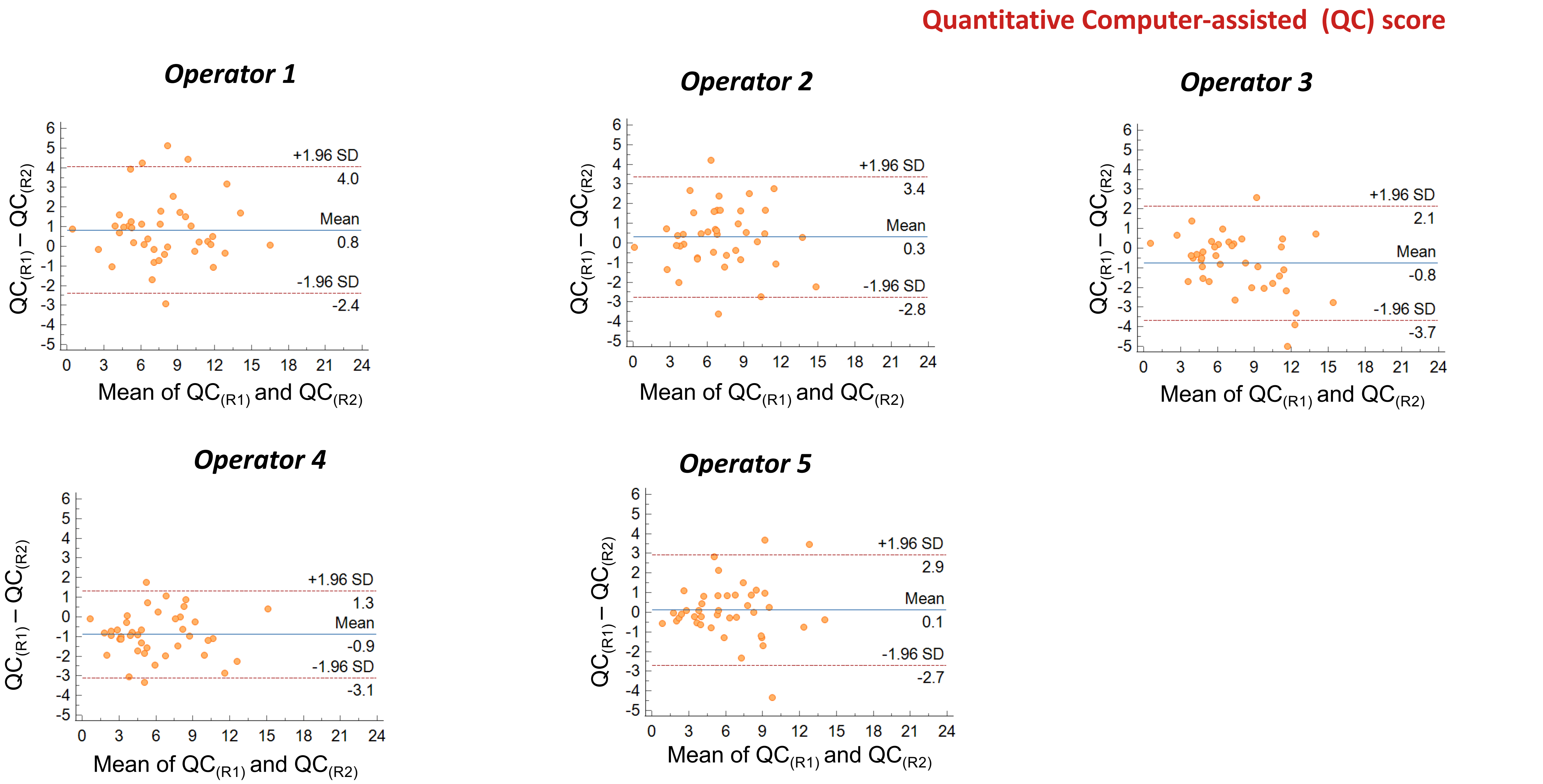

Figure S3. Bland-Altman plots of the intra-operator differences in the estimate of calcification scores between repetitions R1 and R2 (pooled data are presented in Figure 2 in the main text)

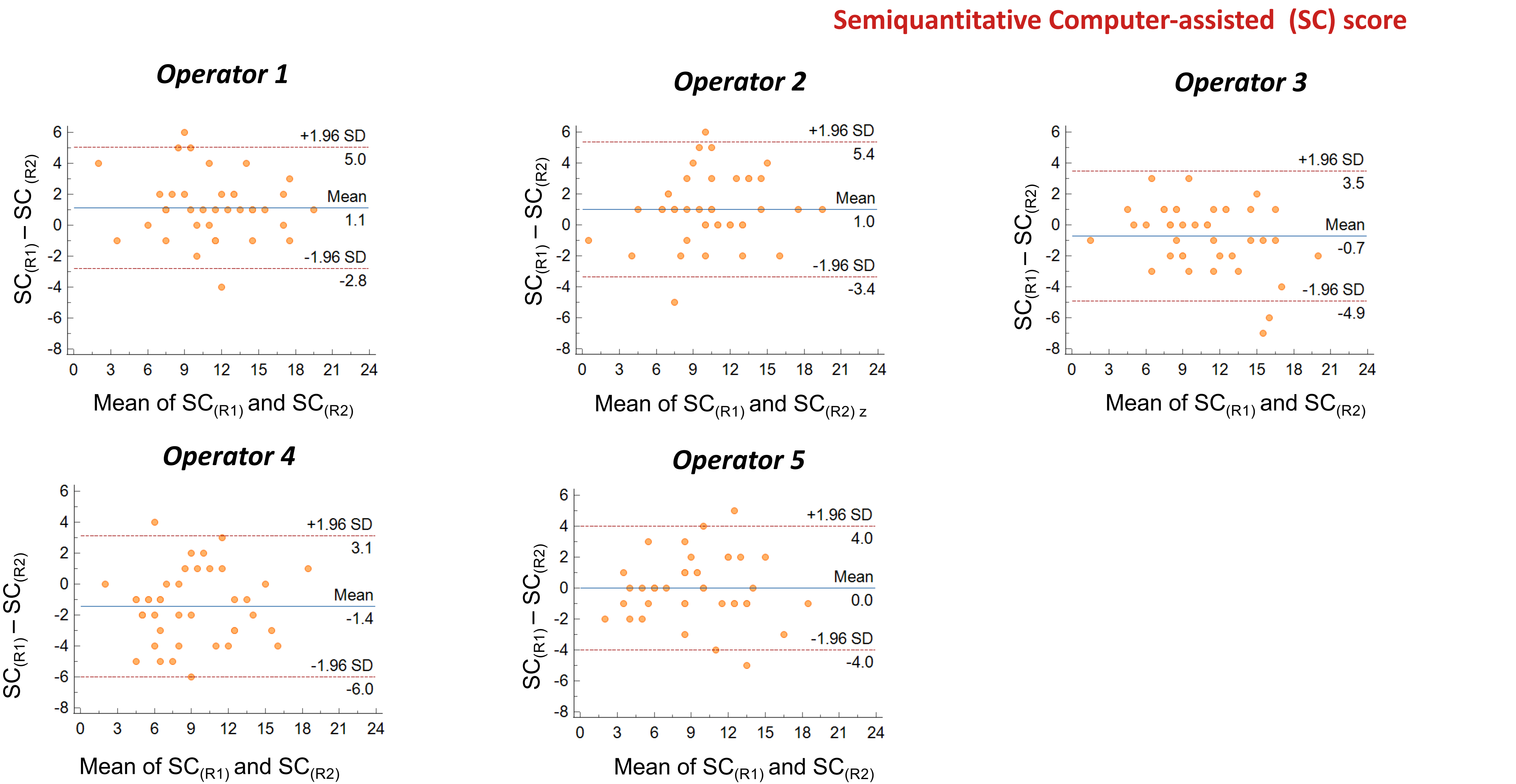

Figure S4. Per-operator regression plots of the estimates of calcification scores in repetitions R1 and R2 (pooled data are presented in Figure 2 in the main text)

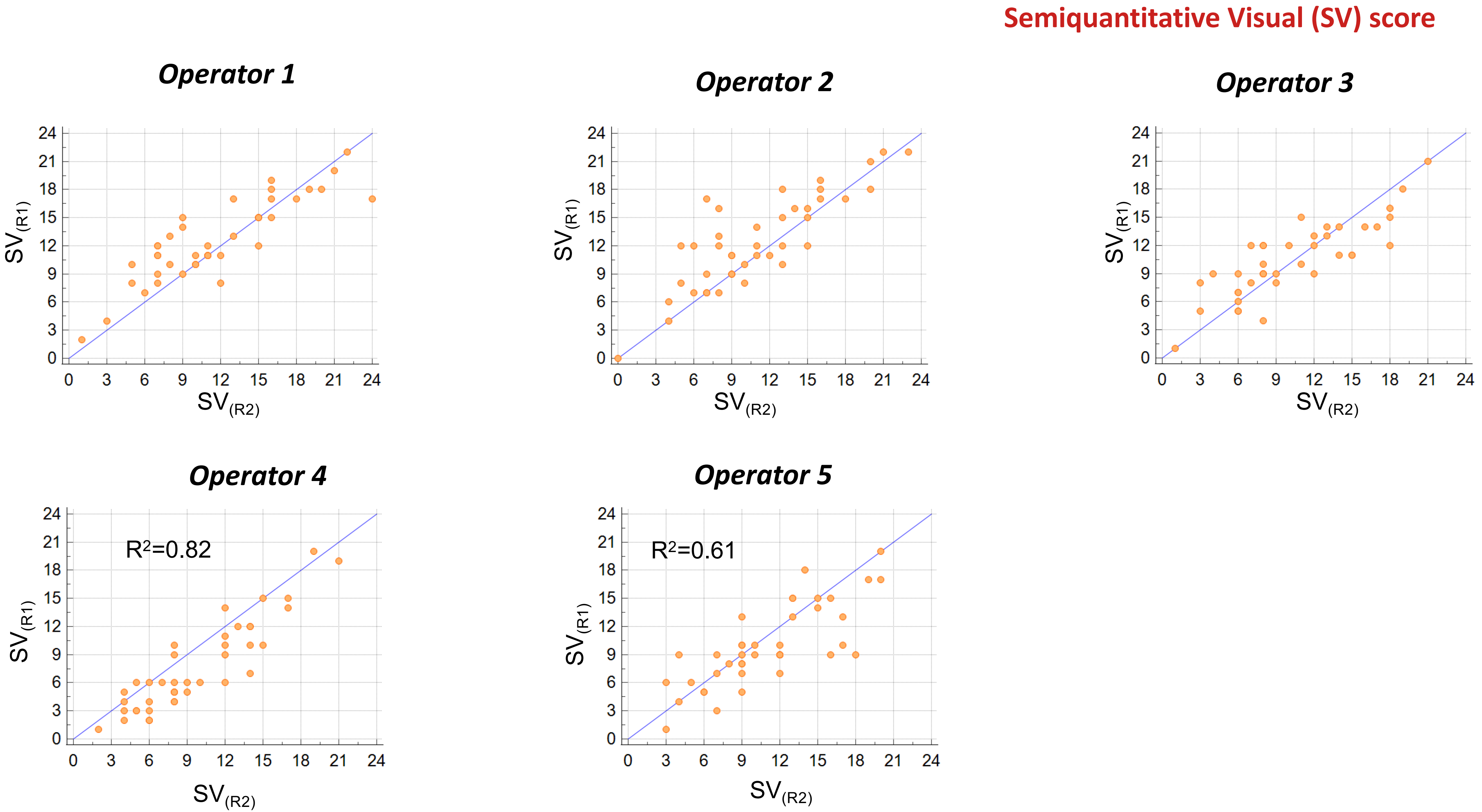

Figure S5. Per-operator regression plots of the estimates of calcification scores in repetitions R1 and R2 (pooled data are presented in Figure 2 in the main text)

Quantitative Computer-assisted (QC) score

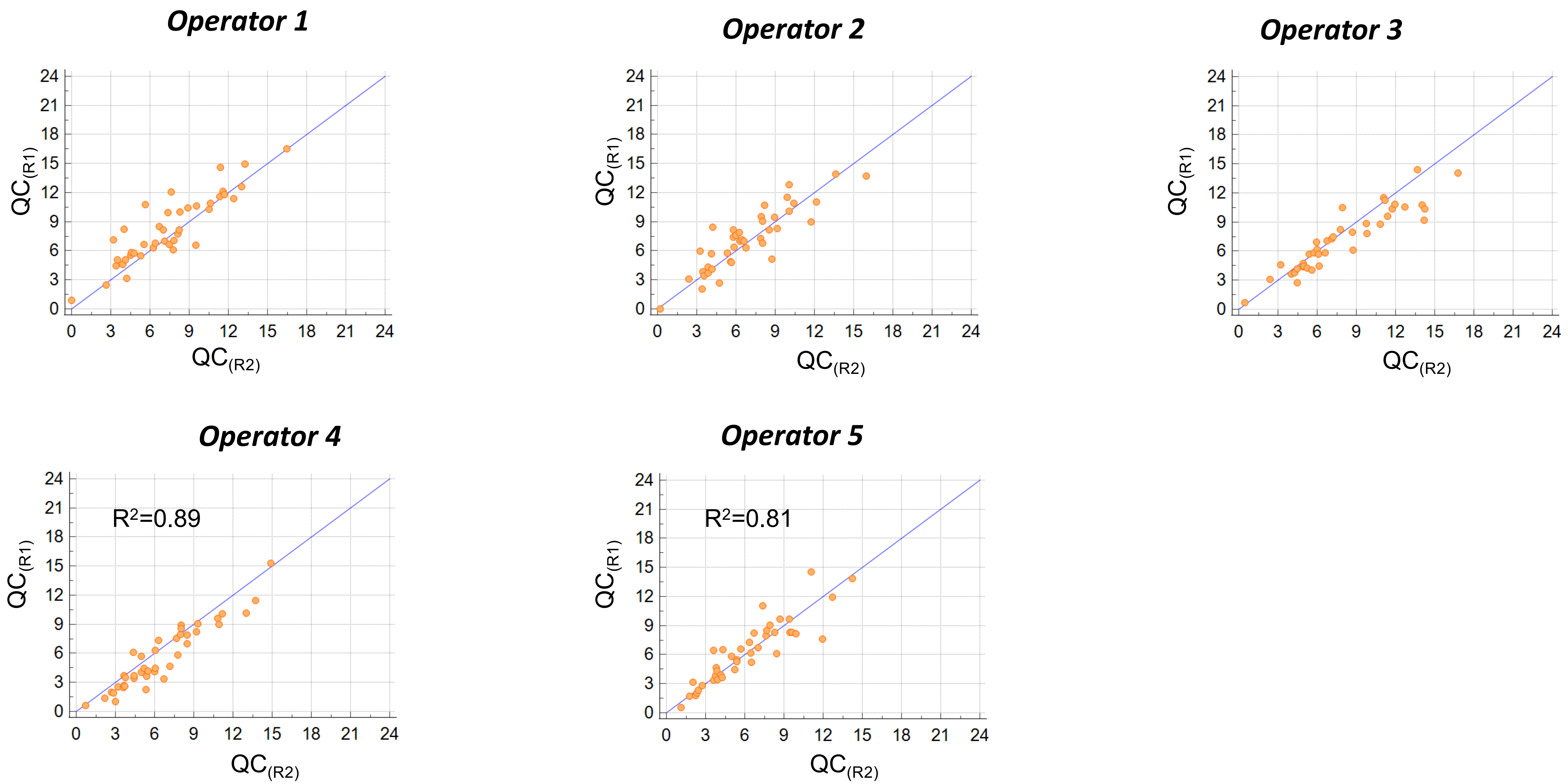

Figure S6. Per-operator regression plots of the estimates of calcification scores in repetitions R1 and R2 (pooled data are presented in Figure 2 in the main text)

Semiquantitative Computer-assisted (SC) score

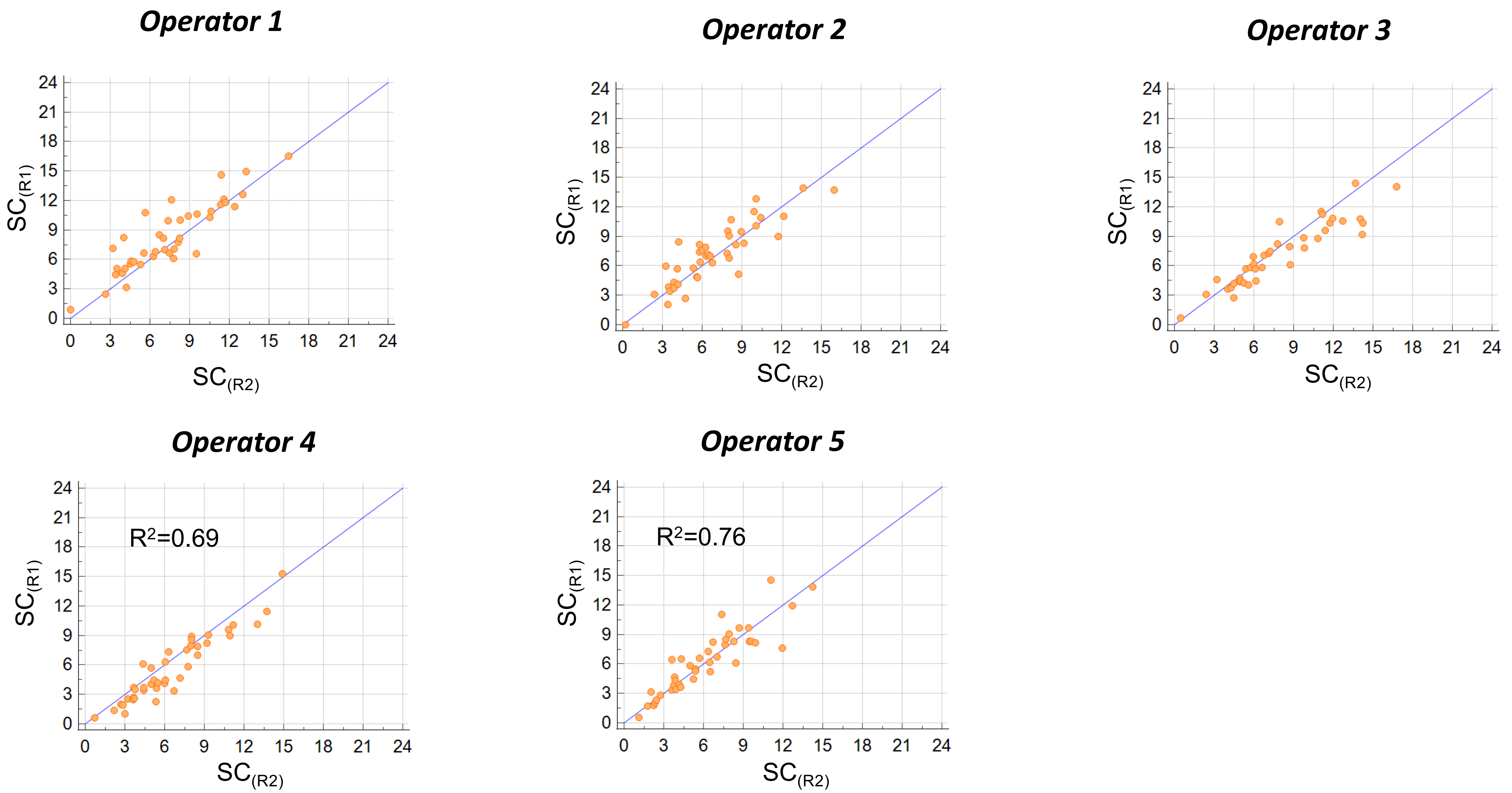

Supplement: Supplementary file 1 [file nutrients-14-04276-s001.zip › nutrients-1922214-supplementary.pdf]
